# Supplementary material for: Venomix: a simple bioinformatic pipeline for identifying and characterizing toxin gene candidates from transcriptomic data
Source: PeerJ. 2018 Jul 31;6:e5361. doi: 10.7717/peerj.5361 (PMC6074769; doi:10.7717/peerj.5361)
Supplement: Supplemental Information 3 [file peerj-06-5361-s003.gz › FinalOutput_E-6/C-type_lectin_6/finaltree.pdf]

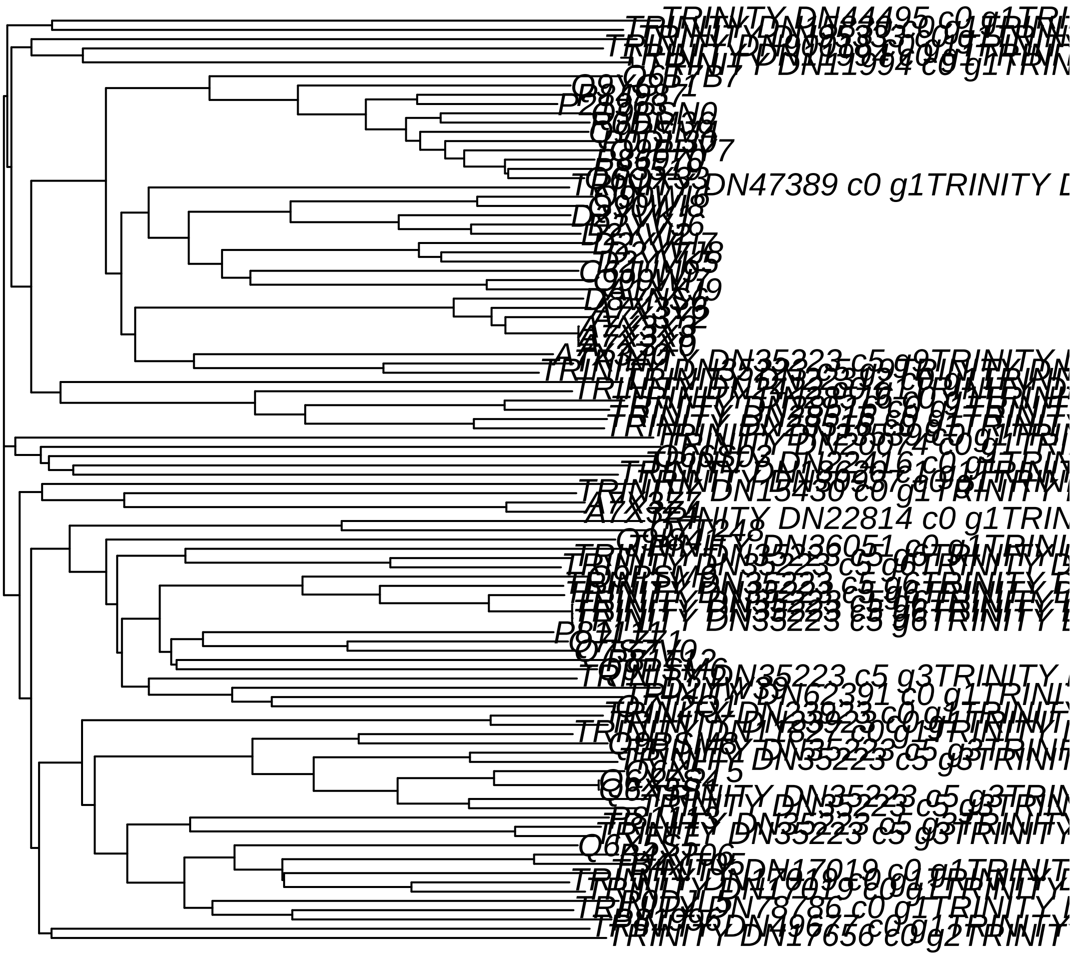
TRINITY DN44495 c0 g1TRI
TRINITY DN11994 c0 g1TRIN
TRINITY DN47389 c0 g1TRINITY D
TRINITY DN15430 c0 g1TRINITY
TRINITY DN22814 c0 g1TRIN
TRINITY DN35223 c5 g6TRINITY
TRINITY DN62391 c0 g1TRIN
TRINITY DN35223 c5 g3TRINIT
TRINITY DN78786 c0 g1TRINITY
TRINITY DN17656 c0 g2TRINIT
